# Trends in Mortality After Incident Hospitalization for Heart Failure Among Medicare Beneficiaries

**DOI:** 10.1001/jamanetworkopen.2024.28964

**Published:** 2024-08-19

**Authors:** Adam S. Vohra, Ali Moghtaderi, Qian Luo, David J. Magid, Bernard Black, Frederick A. Masoudi, Vinay Kini

**Affiliations:** 1 Division of Cardiology, Weill Cornell Medical College, New York, New York; 2 Department of Health Policy and Management, George Washington University, Washington, DC; 3 Division of Cardiology, University of Colorado Anschutz Medical Campus, Englewood; 4 Pritzker School of Law and Kellogg School of Management, Northwestern University, Chicago, Illinois; 5 Ascension Health, St Louis, Missouri

## Abstract

**Importance:**

Despite advances in treatment and care quality for patients hospitalized with heart failure (HF), minimal improvement in mortality has been observed after HF hospitalization since 2010.

**Objective:**

To evaluate trends in mortality rates across specific intervals after hospitalization.

**Design, Setting, and Participants:**

This cohort study evaluated a random sample of Medicare fee-for-service beneficiaries with incident HF hospitalization from January 1, 2008, to December 31, 2018. Data were analyzed from February 2023 to May 2024.

**Main Outcomes and Measures:**

Unadjusted mortality rates were calculated by dividing the number of all-cause deaths by the number of patients with incident HF hospitalization for the following periods: in-hospital, 30 days (0-30 days after hospital discharge), short term (31 days to 1 year after discharge), intermediate term (1-2 years after discharge), and long term (2-3 years after discharge). Each period was considered separately (ie, patients who died during one period were not counted in subsequent periods). Annual unadjusted and risk-adjusted mortality ratios were calculated (using logistic regression to account for differences in patient characteristics), defined as observed mortality divided by expected mortality based on 2008 rates.

**Results:**

A total of 1 256 041 patients (mean [SD] age, 83.0 [7.6] years; 56.0% female; 86.0% White) were hospitalized with incident HF. There was a substantial decrease in the mortality ratio for the in-hospital period (unadjusted ratio, 0.77; 95% CI, 0.67-0.77; risk-adjusted ratio, 0.74; 95% CI, 0.71-0.76). For subsequent periods, mortality ratios increased through 2013 and then decreased through 2018, resulting in no reductions in unadjusted postdischarge mortality during the full study period (30-day mortality ratio, 0.94; 95% CI, 0.82-1.06; short-term mortality ratio, 1.02; 95% CI, 0.87-1.17; intermediate-term mortality ratio, 0.99; 95% CI, 0.79-1.19; and long-term mortality ratio, 0.96; 95% CI, 0.76-1.16) and small reductions in risk-adjusted postdischarge mortality during the full study period (30-day mortality ratio, 0.88; 95% CI, 0.86-0.90; short-term mortality ratio, 0.94; 95% CI, 0.94-0.95; intermediate-term mortality ratio, 0.94; 95% CI, 0.92-0.95; and long-term mortality ratio, 0.95; 95% CI, 0.93-0.96).

**Conclusions and Relevance:**

In this study of Medicare fee-for-service beneficiaries, there was a substantial decrease in in-hospital mortality for patients hospitalized with incident HF from 2008 to 2018, but little to no reduction in mortality for subsequent periods up to 3 years after hospitalization. These results suggest opportunities to improve longitudinal outpatient care for patients with HF after hospital discharge.

## Key Points

**Question:**

What were the trends in mortality rates at specific intervals after hospitalization for heart failure?

**Findings:**

In this cohort study of 1 256 041 Medicare fee-for-service beneficiaries with incident heart failure hospitalization from 2008 to 2018, substantial decreases in mortality were found for the in-hospital period. Little to no reduction in mortality was found for any postdischarge period after hospitalization.

**Meaning:**

These findings suggest that there may be significant opportunities to improve care of patients with heart failure in longitudinal outpatient care after hospital discharge.

## Teaser

This cohort study examines risk-adjusted short-term, intermediate-term, and long-term mortality among Medicare fee-for-service beneficiaries hospitalized for heart failure.

## Introduction

Mortality after hospitalization for heart failure (HF) decreased steadily from approximately 2000 to 2010.^1,2,3^ However, several studies have shown that mortality improvements slowed after approximately 2011 despite continued medical advances in pharmacologic and intervention therapies for the treatment of HF during this time.^4,5,6,7,8,9^ The reasons for the slowing of HF mortality reductions after 2011 are unclear. Prior studies have suggested that reasons might include slow uptake of guideline-directed technologies and medical therapies,^10,11^ nonadherence to HF medications,^12^ and/or paucity of effective therapies in patients with HF with preserved ejection fraction.^13,14,15^ However, each of these potential causes may affect mortality at specific periods after hospitalization for incident HF in different ways. Although some studies have examined trends in short-term (eg, in-hospital or 30 days after discharge) mortality for patients with HF,^11,16,17^ little is known about mortality trends for separate intervals further after HF hospitalization. Furthermore, most prior studies^1,2,9^ have not separately analyzed patients with a first (incident) HF hospitalization, a time when multiple new therapies may be initiated and advanced treatment options may be considered. Understanding these trends could help determine the periods during which the greatest mortality improvements (or lack of improvements) have occurred and efforts to improve care quality should be focused among patients with an incident HF hospitalization.

Accordingly, we used a large sample of Medicare fee-for-service beneficiaries hospitalized with incident HF between 2008 and 2018 to examine trends in risk-adjusted mortality for separate periods: in-hospital, 30-day (0-30 days after discharge), short-term (31 days to 1 year after discharge), intermediate-term (1-2 years after discharge), and long-term (2-3 years after discharge) periods. By considering each period separately (excluding patients who died in a prior period in the mortality assessment of subsequent periods), we may better understand where interventions to improve mortality after HF hospitalization might have the greatest impact.

## Methods

### Data Source

The George Washington University institutional review board determined that this cohort study was exempt from review and the need for informed consent because it did not involve individually identifiable data. This study adheres to the Strengthening the Reporting of Observational Studies in Epidemiology (STROBE) guideline. We used a database of longitudinal Medicare fee-for-service claims (Parts A and B) covering a random sample of approximately 75% of all Medicare fee-for-service patients with an incident HF hospitalization from January 1, 2008, to December 31, 2018. All beneficiaries had at least 3 years’ worth of data to identify prior HF hospitalizations and were enrolled continuously in Medicare Parts A and B from (and including) the month that is 36 months before the date of the index admission and moving forward. The database contains claims through 2019 to allow for 1 year of follow-up for patients hospitalized with incident HF in 2018 (eAppendix in Supplement 1).

### Study Population and Variables

We used *International Classification of Diseases, Ninth Revision, Clinical Modification* (*ICD-9-CM*) and *International Statistical Classification of Diseases and Related Health Problems, Tenth Revision* (*ICD-10*) codes to identify the first hospitalization for HF for all patients between January 1, 2008, and December 31, 2018. We used a minimum look-back period of 3 years to exclude patients who had a prior hospitalization with HF as a primary diagnosis. We also excluded patients who left the hospital against medical advice or were discharged to hospice because they may not have received standard inpatient or outpatient care. We used *ICD-9-CM* and *ICD-10-CM* codes (eTable 1 in Supplement 1) to categorize patients with HF with reduced ejection fraction (HFrEF) and preserved ejection fraction (HFpEF).

The Medicare Master Beneficiary Summary File was used to identify patient demographic characteristics, including race and ethnicity, as well as date of death. The Chronic Conditions Warehouse algorithm provided by the Centers for Medicare & Medicaid Services (version date February 2023) and the Elixhauser Comorbidity Index were used to identify comorbid conditions. Among these potential comorbidities, we chose those most clinically relevant to outcomes for patients with incident HF based on prior literature,^16^ including atrial fibrillation, Alzheimer dementia, metastatic cancer, chronic kidney disease, chronic obstructive pulmonary disease, depression, diabetes, ischemic heart disease, hypertension, peripheral vascular disease, and liver disease (eTable 2 in Supplement 1).

### Covariates and Outcomes

The primary outcome was all-cause mortality during the following periods: in-hospital, 30 days (0-30 days after discharge), short term (31 days to 1 year after discharge), intermediate term (1-2 years after discharge), and long term (2-3 years after discharge). Patients who died in a prior period were not counted in the assessment of mortality for subsequent periods. We separately examined trends for patients with HFrEF and HFpEF. We excluded patients with unspecified HF in this subgroup analysis; *ICD-9-CM* and *ICD-10-CM* diagnosis codes used are provided in the eAppendix in Supplement 1. Because our dataset contains claims through the end of 2019, we did not report intermediate- or long-term mortality for patients with incident HF in 2018 or long-term mortality for patients with incident HF in 2017.

Prior studies have demonstrated a secular increase in the coding of comorbid conditions on billing claims during the study period, which may be due to (1) changes in diagnostic criteria for certain conditions (eg, reductions in the glomerular filtration rate threshold to diagnose chronic kidney disease), (2) changes in coding practices to reflect increases in patient severity of illness (ie, upcoding), or (3) a true increase in the population prevalence of comorbid conditions.^18,19^ Furthermore, the Medicare Advantage program grew during the study period and could have led to changes in the Medicare fee-for-service population during the study period. Thus, we (1) assessed both unadjusted and risk-adjusted mortality rates, (2) used the Medicare 5% sample to assess trends by age and sex in the Medicare fee-for-service and Medicare Advantage populations (eTable 3 in Supplement 1), and (3) performed a sensitivity analysis to address potential changes in the study population over time.

### Statistical Analysis

Characteristics of patients with HF for selected years spanning the study period (2008, 2013, and 2018), including age, sex, race and ethnicity, and comorbidities, were compared. Two-tailed *t* tests or χ^2^ tests were used for comparing the 2008 and 2018 cohort. Annual unadjusted mortality rates were calculated by dividing the total number of deaths within each period by the total number of patients with incident HF in the base year, after excluding patients who died in prior periods. We adopted the methods used by Krumholz et al^20^ to estimate mortality ratios adjusted for patient demographic and comorbid conditions. This analysis was conducted at the patient level with hospital random effects. Data from 2008 were used to fit a mixed-effects generalized linear model with hospital random effects and a logit link function to model mortality for each HF year and period since HF hospitalization. The outcome for each period is a binary variable that takes the value of 1 if a patient died within that period and 0 otherwise. Using parameters estimated for patients with incident HF hospitalization in the base year, we calculated expected mortality for in-hospital and postdischarge periods between 2009 and 2018. The risk-adjusted mortality ratios were obtained by dividing the observed mortality by the expected mortality. We used bootstrapping to estimate 95% CIs for mortality ratios. A 2-sided *P* < .05 was considered to be statistically significant. Data were analyzed between February 2023 and May 2024.

### Sensitivity Analysis

We performed 3 sensitivity analyses. First, we excluded patients with a prior outpatient diagnosis of HF to assess whether results differed without patients who were potentially initiated on HF treatments in the outpatient setting. Second, we included length of stay as a covariate in our models because hospitals were incentivized over time to reduce length of stay, which could lead to reductions in in-hospital mortality that are not necessarily reflective of improvements in care. Third, we used an alternate adjustment method that (1) allowed the association between demographic and comorbid conditions and mortality to differ in each year, which could help account for potential increases in coding of comorbid conditions in later years and potential changes in the Medicare fee-for-service population over time, and (2) used a linear probability regression with hospital fixed effects (which fully control for hospital characteristics) rather than a logit model with hospital random effects. Full details are provided in the eAppendix in Supplement 1.

## Results

### Patient Characteristics

We identified 1 256 041 patients hospitalized for incident HF between 2008 and 2018. The mean (SD) patient age was 83.0 (7.6) years (82.9 [7.4] years in 2008 and 82.6 [8.0] years in 2018, *P* < .001) (Table 1). The patients were 56.0% female and 44.0% male; 1.4% were Asian or Pacific Islander, 7.5% were Black, 3.6% were Hispanic, 86.0% were White, and 1.5% were of other or unknown race, including American Indian or Alaska Native and multiple races. The proportion of male patients increased from 42.2% to 45.0% during the study period (*P* < .001), and the mean (SD) number of comorbidities increased from 3.3 (1.8) in 2008 to 3.8 (1.9) in 2018 (*P* < .001). There was a small decrease in the number of patients with incident HF in 2016 after the transition from *ICD-9-CM* to *ICD-10-CM* that recovered to pretransition numbers in 2017; the trend toward increased HF diagnosis specificity accelerated during the transition (eTable 4 in Supplement 1).

**Table 1.** Characteristics of Patients Hospitalized for Incident Heart Failure in 2008, 2013, and 2018

Characteristics	Participants, %^a^			*P* value^b^
	2008 (n = 125 791)	2013 (n = 107 490)	2018 (n = 120 975)	
Age, mean (SD), y	82.9 (7.4)	83.1 (7.7)	82.6 (8.0)	<.001
Sex				
Female	57.8	56.1	55.0	<.001
Male	42.2	43.9	45.0	
Race and ethnicity				<.001
Asian or Pacific Islander	1.1	1.4	1.6	<.001
Black	7.3	7.4	7.7	<.001
Hispanic	3.4	3.5	3.8	<.001
White	87.4	86.7	85.4	<.001
Other or unknown^c^	0.8	0.9	1.5	<.001
No. of comorbidities, mean (SD)	3.29 (1.76)	3.67 (1.84)	3.84 (1.87)	<.001
Comorbidities				
Atrial fibrillation	28.9	34.9	26.9	<.001
Alzheimer dementia	6.0	5.4	4.6	<.001
Metastatic cancer	2.8	2.7	3.1	<.001
Chronic kidney disease	25.8	36.6	49.4	<.001
COPD and bronchitis	40.2	45.2	43.5	<.001
Depression	15.7	20.3	23.3	<.001
Diabetes	38.9	41.7	43.6	<.001
Ischemic heart disease	49.1	50.2	48.6	.03
Hypertension	80.8	85.8	88.2	<.001
Peripheral vascular disease	35.1	38.0	45.4	<.001
Liver disease	5.0	5.9	7.3	<.001
Heart failure type				
Reduced ejection fraction	26.1	38.7	43.1	<.001
Preserved ejection fraction	22.9	41.8	53.1	<.001
Unspecified	51.0	19.5	3.8	<.001
Prior outpatient heart failure diagnosis	50.0	50.2	49.4	.04
Length of stay, mean (SD), d	5.57 (4.46)	5.33 (4.10)	5.17 (3.97)	<.001

Abbreviation: COPD, chronic obstructive pulmonary disease.

^a^ Patients for selected years in the study are compared here and do not include all patients identified.

^b^ *P* values for 2018 vs 2008 pairwise differences using a 2-tailed χ^2^ or *t* test.

^c^ Other includes American Indian or Alaska Native or multiple races.

### Overall HF Mortality by Period

Unadjusted mortality rates for each year and period are presented in Table 2. In 2008, the absolute mortality rates were 3.6% for the in-hospital period, 6.7% for the 30-day period, 26.4% for the short-term period, 14.7% for the intermediate-term period, and 11.1% for the long-term period.

**Table 2.** Unadjusted Mortality Rates (95% CIs) for Patients Hospitalized With Incident Heart Failure, 2008-2018^a^

Year	In-hospital	30 Days (0-30 d)	Short term (31 d-1 y)	Intermediate term (1-2 y)	Long term (2-3 y)
2008	3.56 (3.46-3.67)	6.73 (6.59-6.87)	26.39 (26.14-26.63)	14.68 (14.47-14.88)	11.13 (10.96-11.31)
2009	3.57 (3.47-3.68)	6.76 (6.62-6.90)	27.10 (26.85-27.34)	14.99 (14.79-15.19)	11.10 (10.93-11.27)
2010	3.46 (3.35-3.57)	6.86 (6.71-7.00)	27.84 (27.58-28.09)	14.88 (14.68-15.09)	11.19 (11.01-11.38)
2011	3.49 (3.38-3.60)	7.13 (6.98-7.28)	28.03 (27.76-28.30)	15.53 (15.32-15.74)	10.97 (10.79-11.15)
2012	3.50 (3.39-3.61)	7.23 (7.08-7.38)	29.10 (28.82-29.38)	14.98 (14.75-15.20)	11.34 (11.14-11.53)
2013	3.39 (3.28-3.50)	7.41 (7.25-7.57)	28.57 (28.30-28.83)	15.54 (15.32-15.76)	10.95 (10.76-11.13)
2014	3.19 (3.09-3.29)	7.11 (6.96-7.26)	28.94 (28.68-29.20)	15.08 (14.87-15.29)	10.90 (10.72-11.09)
2015	3.12 (3.01-3.22)	7.05 (6.90-7.20)	28.39 (28.13-28.65)	15.07 (14.85-15.29)	11.08 (10.90-11.26)
2016	2.89 (2.78-2.99)	6.86 (6.71-7.01)	28.31 (28.04-28.58)	14.88 (14.66-15.10)	10.71 (10.51-10.90)
2017	2.88 (2.78-2.97)	6.72 (6.57-6.86)	27.74 (27.48-28.00)	14.56 (14.37-14.76)	NA
2018	2.73 (2.64-2.83)	6.32 (6.19-6.45)	26.95 (26.71-27.19)	NA	NA

Abbreviation: NA, not applicable.

^a^ The mortality rates were obtained by dividing the total number of beneficiaries who died during each period by the total number of patients with heart failure.

**Figure 1.**
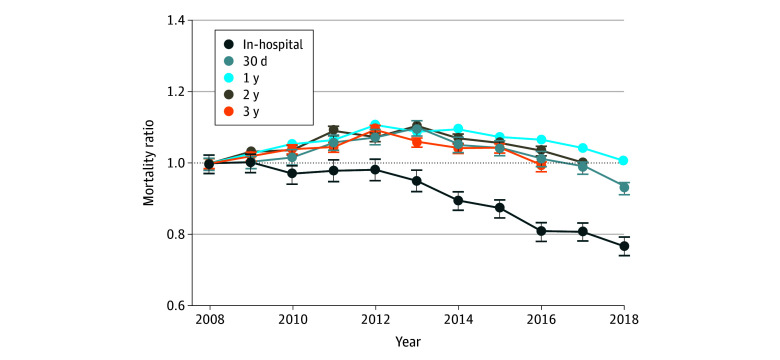
Unadjusted Mortality Ratios (Compared With 2008) for Patients With Heart Failure for Each Study Period Sample includes all Medicare fee-for-service patients with an incident heart failure hospitalization from 2008 through 2018. Mortality ratios are provided as sample means (SDs) in each year normalized by the equivalent rate in 2008. Errors bars indicate SDs.

**Figure 2.**
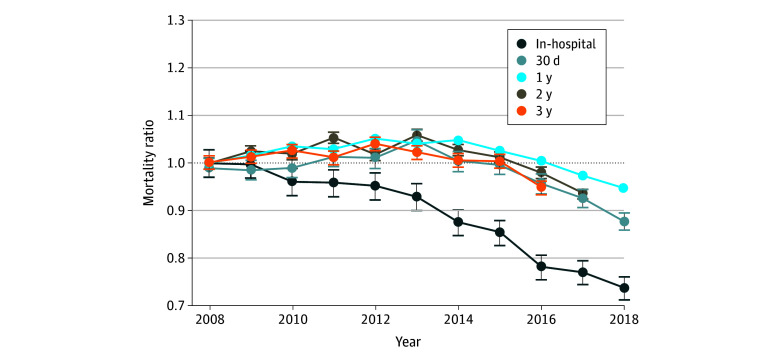
Risk-Adjusted Mortality Ratios (Compared With 2008) for Patients With Heart Failure for Each Study Period Sample includes all Medicare fee-for-service patients with an incident heart failure hospitalization from 2008 through 2018. We used 2008 as a base year and the estimated parameters from this year to estimate expected mortality for each period in later years. The ratio of actual to expected mortality for each year and each period is shown, with bootstrapped 95% CIs (error bars).

Annual unadjusted mortality ratios (compared with 2008) for each period are shown in Figure 1, and risk-adjusted mortality ratios are shown in Figure 2. There was a sustained and substantial decrease in the mortality ratio for the in-hospital period (unadjusted mortality ratio, 0.77; 95% CI, 0.67-0.77; risk-adjusted ratio, 0.74; 95% CI, 0.71-0.76). For subsequent periods, mortality ratios increased through approximately 2013 and then decreased through 2018. For the full study period, there were no significant reductions in unadjusted mortality (30-day mortality ratio, 0.94; 95% CI, 0.82-1.06; short-term mortality ratio, 1.02; 95% CI, 0.87-1.17; intermediate-term mortality ratio, 0.99; 95% CI, 0.79-1.19; and long-term mortality ratio, 0.96; 95% CI, 0.76-1.16) and small reductions in risk-adjusted mortality (30-day mortality ratio, 0.88; 95% CI, 0.86-0.90; short-term mortality ratio, 0.94; 95% CI, 0.94-0.95; intermediate-term mortality ratio, 0.94; 95% CI, 0.92-0.95; and long-term mortality ratio, 0.95; 95% CI, 0.93-0.96). Numerical risk-adjusted mortality ratios are provided in eTable 5 in Supplement 1. Cumulative risk-adjusted mortality ratios are also provided in eFigure 1 in Supplement 1.

### HFrEF and HFpEF Mortality

**Figure 3.**
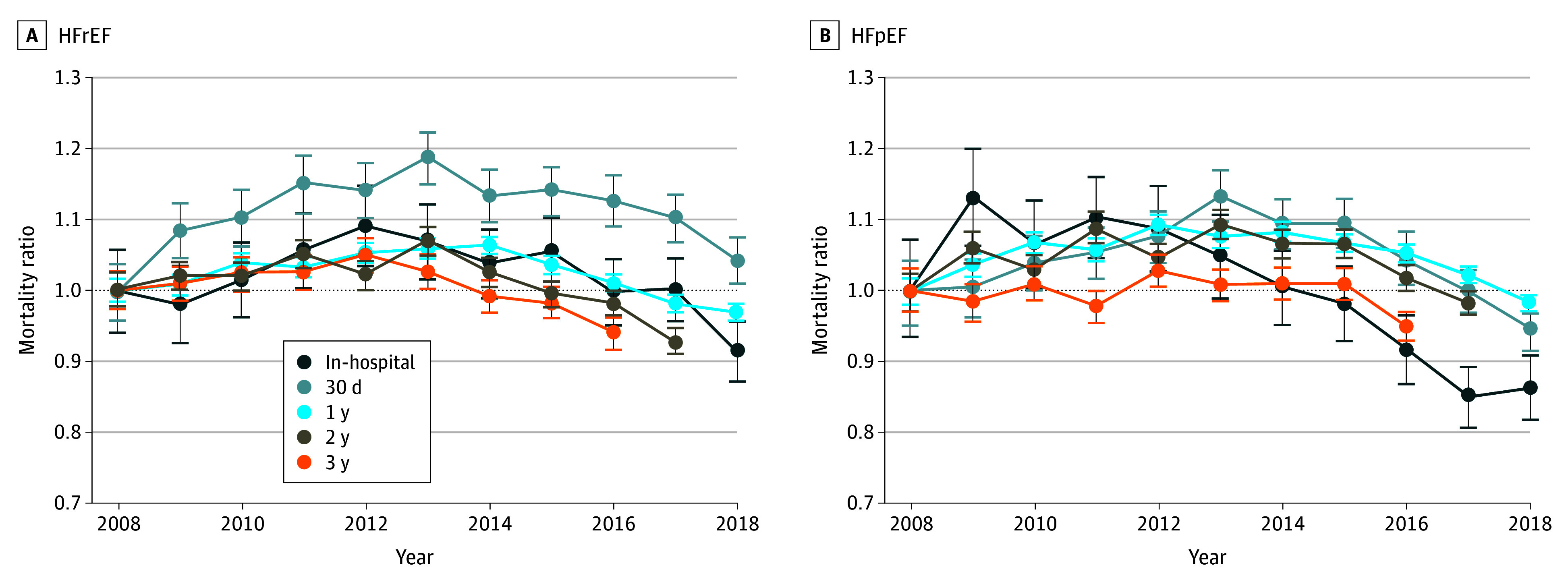
Risk Standardized Mortality Ratios (Compared With 2008) for Patients With Heart Failure With Reduced Ejection Fraction (HFrEF) and Heart Failure With Preserved Ejection Fraction (HFpEF) for Each Study Period Sample includes all Medicare fee-for-service patients with an incident heart failure hospitalization from 2008 through 2018. We used 2008 as a base year and the estimated parameters from this year to estimate expected mortality for each period in later years. The ratio of actual to expected mortality for each year and each period is shown, with bootstrapped 95% CIs (error bars).

The proportion of patients with a diagnosis of unspecified HF decreased from 51.0% in 2008 to 3.8% in 2018 (eFigure 2 in Supplement 1). Among patients with HF of a specified type, the proportion of patients with HFrEF relative to HFpEF decreased from 1.1 in 2008 to 0.8 in 2018. We observed mostly similar overall risk-adjusted mortality rates for each period for patients with HFrEF vs HFpEF (eFigure 3 in Supplement 1). There was a modestly larger but not statistically significant reduction in risk-adjusted mortality for the in-hospital period for patients with HFpEF (mortality ratio, 0.86; 95% CI, 0.82-0.91) compared with patients with HFrEF (mortality ratio, 0.92; 95% CI, 0.87-0.96) and a steeper mortality decrease observed for patients with HFpEF for each postdischarge period in the later years of the study (Figure 3). Numerical risk-adjusted mortality ratios for each year for patients with HFrEF and HFpEF are provided in eTables 6 and 7 in Supplement 1.

### Sensitivity Analysis

Sensitivity analyses that excluded patients with prior outpatient diagnoses of HF and adjusted for length of stay as a covariate showed similar trends in mortality for each period to those in the risk-adjusted model. Sensitivity analyses that allowed associations between comorbidities and mortality to vary by year and used a linear probability model with hospital fixed effects also showed similar results to the risk-adjusted model. Full results of these analyses are provided in eFigures 3 to 5 in Supplement 1.

## Discussion

### Summary of Findings and Extension of Prior Literature

In this study of fee-for-service Medicare beneficiaries hospitalized with incident HF from 2008 to 2018, we found a substantial decrease in in-hospital mortality during the study period in both unadjusted and risk-adjusted analyses. However, for all postdischarge periods studied, we observed small increases in mortality in the first half of the sample period followed by small decreases in mortality during the later years of the study. The small postdischarge mortality decreases in the later years of the study were slightly stronger in risk-adjusted analyses than in unadjusted analyses, reflecting the secular increase in the coding of comorbid conditions during the study period. Thus, for the overall study period, we found at best little improvement (using risk-adjusted analyses) and at worst no improvement (using unadjusted analyses) in postdischarge mortality for patients with incident HF through 3 years after discharge.

Prior studies have assessed cumulative HF mortality during certain time periods (eg, 30 days or 1 year)^1,16,17^ and have shown decreases in in-hospital mortality but possible increases in 30-day mortality (0-30 days after discharge) from approximately 2010 to 2013.^21,22,23^ One recent study found that HF-related mortality increased from 2012 to 2021 but was limited by reliance on death certificate data, which may misattribute some deaths.^9^ Our study advances the current understanding of HF mortality rates in the following ways. First, we evaluated mortality changes during specific follow-up periods and studied a longer postdischarge period (ie, up to 3 years). Second, we focused on incident HF hospitalization, a time when guidelines recommend consideration and initiation of multiple HF therapies at once. Third, we use both unadjusted and risk-adjusted analyses to fully understand mortality regardless of potential changes in coding practices. Our results suggest that future efforts to improve HF care that are focused on longitudinal outpatient follow-up may represent the greatest opportunity for patient benefit.

### Potential Explanations for Observed Trends

During the past 2 decades, there have been medical advances throughout the spectrum of management for HF, including new pharmacologic treatments shown to improve outcomes, new technologies to improve heart function, and new devices to replace failing hearts. Randomized clinical trials have shown that many of these treatments significantly reduce mortality for patients with HF.^4,5,6,24^ One study showed that treatment with comprehensive disease-modifying therapy for patients with HFrEF could extend survival by 1.4 years for an 80-year-old patient.^7^ However, our study suggests a discrepancy between clinical trial findings and mortality rates for Medicare patients who survive an incident HF hospitalization. Although our study was not designed to understand the causal mechanisms of mortality trends, the small increases in postdischarge mortality during the early years of our study could be due to several factors, including higher severity of illness or unintended consequences of health care payment policies.^17,22,25^ The subsequent reductions in postdischarge mortality starting in approximately 2013 are likely also multifactorial but potentially due to better use of guideline-directed medical therapies or greater dissemination of new treatments for patients with HF.

In recent years, many efforts to enact guideline-directed medical therapy and other treatments for HF have focused on the hospital setting.^26,27^ However, our study suggests that these interventions have not led to significant longer-term mortality improvements, possibly due to barriers to implementation of quality improvement efforts in the outpatient setting. For example, in addition to guideline-directed medical therapy for HF, prior literature has also highlighted low rates of participation in cardiac rehabilitation,^28,29,30^ underuse of cardiac resynchronization therapy,^31^ and possible delayed referral to advanced HF specialists.^32^ Cardiac rehabilitation has been shown to increase exercise capacity, reduce hospitalization, and in some cases decrease all-cause mortality.^30,33,34^ However, rates of participation have remained low at approximately one-third of patients.^35^ Focusing efforts on improving the use of evidence-based treatments by identifying and addressing barriers—such as low referral rates, geographic proximity to care, affordability of medications, and increased health care literacy—in the outpatient setting has the potential to improve longer-term outcomes for patients with HF.^35^ The small mortality improvements observed for long-term mortality in the later years of our study may reflect greater recognition and use of these programs and treatments.

### Differences Between Patients With HFrEF and HFpEF 

The proportion of patients classified as having HFrEF or HFpEF increased during the study period, reflecting better specificity in coding. The proportion of patients with HFpEF relative to HFrEF also increased, likely reflecting increasing clinical recognition of the syndrome HFpEF. Although our study suggests a slightly larger decrease in in-hospital mortality for patients with HFpEF compared with patients with HFrEF, it was not designed to evaluate the causes of mortality findings. The improvements in in-hospital mortality for patients with HFpEF may reflect improvements in care for noncardiovascular conditions, such as diabetes and pulmonary diseases, for which patients with HFpEF typically have a larger burden compared with patients with HFrEF.^36,37^ A prior study found that with increasing age, a smaller proportion of deaths in patients with HF are due to cardiovascular causes, and for HFpEF specifically, for patients older than 65 years, less than half are due to cardiovascular causes.^38^ Further study is needed to verify and understand potential differences in HFrEF and HFpEF mortality.

### Limitations

Our study has several limitations. First, our mortality risk adjustment method could have been affected by changes in coding over time, including the switch from *ICD-9-CM* to *ICD-10-CM* and upcoding of comorbid conditions. This may have the effect of the population appearing “sicker” over time, resulting in falsely low estimates of risk-adjusted mortality. To address this risk, we reported results of unadjusted analyses and performed sensitivity analyses in which we risk adjusted within each year rather than across years (Supplement 1). Second, our study relies on claims data rather than clinical data, which could enable better cohort characterization and risk adjustment (such as by including smoking status) as well as better identify HF subtypes.^39,40^ Third, with our data we are unable to assess the reasons underlying the differences in mortality trends observed across the study period. Fourth, there was increasing recognition of HFpEF during the years of our study, but our data are limited by low diagnosis specificity, particularly during the early years of the sample period. Further study using clinical data is needed to better understand differences in mortality trends between patients with HFpEF and HFrEF. Finally, our study population is limited to Medicare fee-for-service beneficiaries. It does not include patients enrolled in Medicare Advantage, a program that grew significantly over time and may have led to changes in the Medicare fee-for-service population during the sample period. Our sensitivity analysis allowing associations between mortality and demographic and comorbid conditions to vary by year helps account for these changes, but residual confounding may be present.

## Conclusions

For patients hospitalized with incident HF from 2008 to 2018, we found a significant decrease in in-hospital mortality but little to no improvements in mortality for subsequent periods through 3 years after discharge. Future efforts focusing on care improvements in the longitudinal outpatient setting may represent the greatest opportunity for patient benefit.

## Notes

Supplement 1. **eAppendix.** Supplemental Methods **eTable 1.** Diagnosis Codes for Heart Failure, Including Preserved and Reduced Ejection Fraction **eTable 2.** Diagnosis Codes for Comorbid Conditions **eTable 3.** Differences in Age and Sex Between Medicare Fee-For-Service and Medicare Advantage Populations in the Medicare 5% Sample **eTable 4.** Annual Number of Incident HF Patients **eTable 5.** Risk-Adjusted Mortality Ratios, Relative to 2008, for Patients Hospitalized With Incident Heart Failure (2008-2018) **eTable 6.** Risk Adjusted Mortality Ratios for HF with Reduced Ejection Fraction Cohort **eTable 7.** Risk Adjusted Mortality Ratios for HF with Preserved Ejection Fraction Cohort **eFigure 1.** Cumulative Risk-Adjusted Mortality Ratios for HF Cohort **eFigure 2.** Risk-Adjusted Mortality Ratios, Excluding Patients with Prior Outpatient HF Diagnosis (sensitivity analysis 1) **eFigure 3.** Annual Proportion of Heart Failure Patients Classified as Reduced or Preserved Ejection Fraction **eFigure 4.** Risk-Adjusted Mortality Ratios, Including Length of Stay as Covariate (sensitivity analysis 2) **eFigure 5.** Risk-Adjusted Mortality Ratios for HF Cohort, Alternative Adjustment Methodology (sensitivity analysis 3)

Supplement 2.
Data Sharing Statement
