# Supplementary material for: Trends in Mortality After Incident Hospitalization for Heart Failure Among Medicare Beneficiaries
Source: JAMA Netw Open. 2024 Aug 19;7(8):e2428964. doi: 10.1001/jamanetworkopen.2024.28964 (PMC11333983; doi:10.1001/jamanetworkopen.2024.28964)
Supplement: Supplement 1. — eAppendix. Supplemental Methods eTable 1. Diagnosis Codes for Heart Failure, Including Preserved and Reduced Ejection Fraction eTable 2. Diagnosis Codes for Comorbid Conditions eTable 3. Differences in Age and Sex Between Medicare Fee-For-Service and Medicare Advantage Populations in the Medicare 5% Sample eTable 4. Annual Number of Incident HF Patients eTable 5. Risk-Adjusted Mortality Ratios, Relative to 2008, for Patients Hospitalized With Incident Heart Failure (2008-2018) eTable 6. Risk Adjusted Mortality Ratios for HF with Reduced Ejection Fraction Cohort eTable 7. Risk Adjusted Mortality Ratios for HF with Preserved Ejection Fraction Cohort eFigure 1. Cumulative Risk-Adjusted Mortality Ratios for HF Cohort eFigure 2. Risk-Adjusted Mortality Ratios, Excluding Patients with Prior Outpatient HF Diagnosis (sensitivity analysis 1) eFigure 3. Annual Proportion of Heart Failure Patients Classified as Reduced or Preserved Ejection Fraction eFigure 4. Risk-Adjusted Mortality Ratios, Including Length of Stay as Covariate (sensitivity analysis 2) eFigure 5. Risk-Adjusted Mortality Ratios for HF Cohort, Alternative Adjustment Methodology (sensitivity analysis 3) [file jamanetwopen-e2428964-s001.pdf]

Supplementary Online Content

Vohra AS, Moghtaderi A, Luo Q, et al. Trends in mortality after incident hospitalization for heart failure among Medicare beneficiaries. *JAMA Netw Open*. 2024;7(8):e2428964. doi:10.1001/jamanetworkopen.2024.28964

eAppendix. Supplemental Methods
eTable 1. Diagnosis Codes for Heart Failure, Including Preserved and Reduced Ejection Fraction
eTable 2. Diagnosis Codes for Comorbid Conditions
eTable 3. Differences in Age and Sex Between Medicare Fee-For-Service and Medicare Advantage Populations in the Medicare 5% Sample
eTable 4. Annual Number of Incident HF Patients
eTable 5. Risk-Adjusted Mortality Ratios, Relative to 2008, for Patients Hospitalized With Incident Heart Failure (2008-2018)
eTable 6. Risk Adjusted Mortality Ratios for HF with Reduced Ejection Fraction Cohort
eTable 7. Risk Adjusted Mortality Ratios for HF with Preserved Ejection Fraction Cohort
eFigure 1. Cumulative Risk-Adjusted Mortality Ratios for HF Cohort
eFigure 2. Risk-Adjusted Mortality Ratios, Excluding Patients with Prior Outpatient HF Diagnosis (sensitivity analysis 1)
eFigure 3. Annual Proportion of Heart Failure Patients Classified as Reduced or Preserved Ejection Fraction
eFigure 4. Risk-Adjusted Mortality Ratios, Including Length of Stay as Covariate (sensitivity analysis 2)
eFigure 5. Risk-Adjusted Mortality Ratios for HF Cohort, Alternative Adjustment Methodology (sensitivity analysis 3)

This supplementary material has been provided by the authors to give readers additional information about their work.

© 2024 Vohra AS et al. JAMA Network Open.

eAppendix. Supplemental Methods

1. Construction of Heart Failure Cohort

We identify from Medicare fee-for-service claims data all beneficiaries hospitalized with primary diagnosis codes of heart failure (HF). We refer to these hospitalizations as index admission. We use the Medicare MedPAR (inpatient) file with any of the following principal diagnosis codes:

> <u>ICD-10 Codes (for hospitalizations on or after Oct 1, 2015):</u> I50, I50.1, I50.2, I50.20, I50.21, I50.22, I50.23, I50.3, I50.30, I50.31, I50.32, I50.33, I50.4, I50.40, I50.41, I50.42, I50.43, I50.8, I50.814, I50.82, I50.83, I50.84, I50.89, I50.9, I09.81, I11.0, I13.0, I13.2
>
> <u>ICD-9 Codes (for hospitalizations before Oct 1, 2015):</u> 398.91, 402.01, 402.11, 402.91, 404.01, 404.03, 404.11, 404.13, 404.91, 404.93, 428, 428.0, 428.1, 428.2, 428.20, 428.21, 428.22, 428.23, 428.3, 428.30, 428.31, 428.32, 428.33, 428.4, 428.40, 428.41, 428.42, 428.43, 428.9

We include the beneficiaries who meet all the following criteria:

(i) Age 68 or older at the time of the index admission.

(ii) Continuously enrolled in both Medicare Part A and Part B from and including the month that is 36 months prior to the date of the index admission.

(iii) The index admission was to a short-term acute care or critical access hospital (excluding special units within these hospitals such as rehab and psychiatric).

(iv) Length of stay for the admission equal or greater than 2.

<u>We Exclude the following as index admissions:</u>

We are interested in the first HF hospitalization (an "incident" HF), we therefore exclude:

(i) Beneficiaries with a hospitalization, prior to the index admission, with a **<u>primary</u> diagnosis** code listed below. For this exclusion, use all available data for each beneficiary even if before 2008.

> <u>ICD-10 Codes (for hospitalizations on or after Oct 1, 2015):</u> I50, I50.1, I50.2, I50.20, I50.21, I50.22, I50.23, I50.3, I50.30, I50.31, I50.32, I50.33, I50.4, I50.40, I50.41, I50.42, I50.43, I50.8, I50.814, I50.82, I50.83, I50.84, I50.89, I50.9 I09.81, I11.0, I13.0, I13.2
>
> <u>ICD-9 Codes (for hospitalizations before Oct 1, 2015):</u> 398.91, 402.01, 402.11, 402.91, 404.01, 404.03, 404.11, 404.13, 404.91, 404.93, 428, 428.0, 428.1, 428.2, 428.20, 428.21, 428.22, 428.23, 428.3, 428.30, 428.31, 428.32, 428.33, 428.4, 428.40, 428.41, 428.42, 428.43, 428.9

(ii) We exclude Beneficiaries with discharge destination code for the index admission which was either against medical advice or hospice. They may not have received standard inpatient or outpatient care.

The detailed descriptions of each diagnosis code used in constructing HF cohort are presented in eTable 1. Diagnosis codes for HFpEF and HFrEF are also reported in eTable 1. Annual numbers of incident HF patients overall and by subtype are reported in eTable 2.

© 2024 Vohra AS et al. JAMA Network Open.

The proportion of Medicare patients enrolled in the Medicare Advantage program grew over the study period and could have led to changes in the Medicare FFS population. We used the 5% sample to assess differences in age and sex between the Medicare FFS and Medicare Advantage populations in eTable 3.

2. Reduced versus Preserved Ejection HF

HF patients can be generally divided into those with reduced ejection fraction (HFrEF) and those with preserved ejection fraction (HpEF), where ejection fraction (EF) is the percent of blood in the main chamber that is ejected with each beat. The classification of HF as with reduced versus preserved ejection fraction is spotty in the early years of our sample, but becomes more complete over time. Additionally, sometimes the diagnosis codes during the hospitalization conflict, or diagnoses conflict between the hospitalization and post-hospitalization period. We developed the following rules to separate HFpEF and HFrEF cases.

1- If the primary discharge diagnosis code is HFrEF or HFpEF, we categorize the visit as HFrEF or HFpEF, regardless of other secondary diagnosis codes.

2- If the primary discharge diagnosis code is unspecified HF, and there is a secondary inpatient diagnosis code of HFrEF or HFpEF, we categorize the visit based on the secondary diagnosis.

3- If the primary discharge diagnosis code is unspecified HF with no secondary inpatient diagnosis code of HFpEF or HFrEF, but there are one or more outpatient claims with diagnosis only of HFrEF or HFpEF within 60 days following discharge, we categorize this visit based on the outpatient diagnosis.

4- If the primary discharge diagnosis code is unspecified HF, with no secondary inpatient diagnosis code of HFpEF or HFrEF and within 60 days after discharge there are either (i) no secondary diagnosis codes for HFpEF or HFrEF; or (ii) codes for both HFpEF and HFrEF, we categorize the visit as unspecified HF.

3. Diagnosis Codes for Comorbid Conditions

We used the Chronic Conditions Warehouse (CCW comorbidities) algorithms provided by the Centers for Medicare and Medicaid Services (CMS) and Elixhauser comorbidity index to identify a variety of comorbid conditions. We used the Medicare claims data in the year prior to the index event to identify the presence of comorbid conditions. We included a separate dummy variable for each condition in our regressions. Conditions that we used for identifying comorbidities are: atrial fibrillation, dementia, metastatic cancer, chronic kidney disease, COPD, Depression, Diabetes, ischemic heart disease, Hypertension, peripheral vascular disease, and Liver disease. eTable 2 presents the corresponding source that we used to identify each comorbid conditions and the references we used for ICD-9 and ICD-10 diagnosis codes.

4. Details of Sensitivity Analyses

We performed three sensitivity analyses. First, we excluded patients with a prior outpatient diagnosis of HF to assess if results differed when accounting for patients who were potentially initiated on HF treatments in the outpatient setting. Second, we included length of stay as a covariate in our models, since hospitals were incentivized over time to reduce length of stay

© 2024 Vohra AS et al. JAMA Network Open.

which could lead to reductions in acute mortality that are not necessarily reflective of improvements in care.

Third, we used an alternative adjustment method to account for two concerns in our main adjustment methodology. The first concern is that our methodology assumes the associations between demographic / comorbid conditions and mortality are stable over time. However, these associations can change over time. This can be either due to changes in the Medicare FFS population over time (such as due to the emergence of Medicare Advantage), improvements in medical innovations and practices that enhances the chance of survival despite the presence of a comorbid condition, or change in the coding practices that indicate higher number of patients with a comorbid condition without any change in underlying population health. The second concern is that we account for clustering effect of hospitals by incorporating hospital random effects that assumes that there is no correlation between the hospital characteristics and other covariates that we employed. This is a strong assumption and the violation of this assumption can lead to biases in other covariate estimates.

We accounted for both of these concerns in an alternative methodology that differs from the main methodology in three important ways: (i) we use the full sample and include an interacted terms between year and age category/sex/comorbid condition dummy variables to allow the association between the comorbid conditions and mortality to vary over time; (ii) we include hospital fixed effects instead of hospital random effects; (iii) since inclusion of hospital fixed effects with logistic regression would result in an incidental parameter problem (since unlike random-effects, fixed-effects should be estimated), we use linear probability model for the alternative strategy.

We first run a linear regression with time increment mortalities as dependent variables, year and demographic/comorbid conditions interaction and hospital fixed-effects as independent variables using the entire sample from 2008-2018. We then used the estimated parameters and average patient characteristics over the entire sample period to estimate the adjusted mortality. We in effect, estimate what is the probability of mortality for a patient with average patient characteristics over the entire sample period. To lend comparability with our main methodology, we normalize all the predicted mortalities by the predicted mortality in 2008.

eFigure 2 presents the risk-adjusted mortality ratios obtained from this alternative specification.

© 2024 Vohra AS et al. JAMA Network Open.

eTable 1- Diagnosis Codes Used in Constructing HF Cohort

Code	Description	HFpEF/HFrEF
**HF ICD-10 Diagnosis Codes**		
I50	Heart failure	Unspecified
I50.1	Left ventricular failure, unspecified	Unspecified
I50.2	Systolic (congestive) heart failure	HFrEF
I50.20	Unspecified systolic (congestive) heart failure	HFrEF
I50.21	Acute systolic (congestive) heart failure	HFrEF
I50.22	Chronic systolic (congestive) heart failure	HFrEF
I50.23	Acute on chronic systolic (congestive) heart failure	HFrEF
I50.3	Diastolic (congestive) heart failure	HFpEF
I50.30	Unspecified diastolic (congestive) heart failure	HFpEF
I50.31	Acute diastolic (congestive) heart failure	HFpEF
I50.32	Chronic diastolic (congestive) heart failure	HFpEF
I50.33	Acute on chronic diastolic (congestive) heart failure	HFpEF
I50.4	Combined systolic (congestive) and diastolic (congestive) heart failure	HFrEF
I50.40	Unspecified combined systolic (congestive) and diastolic (congestive) heart failure	HFrEF
I50.41	Acute combined systolic (congestive) and diastolic (congestive) heart failure	HFrEF
I50.42	Chronic combined systolic (congestive) and diastolic (congestive) heart failure	HFrEF
I50.43	Acute on chronic combined systolic (congestive) and diastolic (congestive) heart failure	HFrEF
I50.8	Other Heart Failure	Unspecified
I50.814	Right Heart Failure due to left heart failure	Unspecified
I50.82	Biventricular heart failure	Unspecified
I50.83	High output heart failure	Unspecified
I50.84	End stage heart failure	Unspecified
I50.89	Other heart failure	Unspecified
I50.9	Heart failure, unspecified	Unspecified
I09.81	Rheumatic heart failure	Unspecified
I11.0	Hypertensive heart disease with heart failure	Unspecified
I13.0	Hypertensive heart and chronic kidney disease with heart failure and stage 1 through stage 4	Unspecified
I13.2	Hypertensive heart and chronic kidney disease with heart failure and with stage 5 chronic kidney disease, or end stage renal disease	Unspecified
**HF ICD-9 Diagnosis Codes**		
398.91	Rheumatic heart failure (congestive); Rheumatic left ventricular failure	Unspecified
402.01	Malignant hypertensive heart disease with heart failure	Unspecified
402.11	Benign hypertensive heart disease with heart failure	Unspecified
402.91	Unspecified hypertensive heart disease with heart failure	Unspecified

© 2024 Vohra AS et al. JAMA Network Open.

Code	Description	HFpEF/HFrEF
404.01	Hypertensive heart and chronic kidney disease, malignant, with heart failure and with chronic kidney disease stage I through stage IV, or unspecified	Unspecified
404.03	Hypertensive heart and chronic kidney disease, malignant, with heart failure and with chronic kidney disease stage V or end stage renal disease	Unspecified
404.11	Hypertensive heart and chronic kidney disease, benign, with heart failure and with chronic kidney disease stage I through stage IV, or unspecified	Unspecified
404.13	Hypertensive heart and chronic kidney disease, benign, with heart failure and chronic kidney disease stage V or end stage renal disease	Unspecified
404.91	Hypertensive heart and chronic kidney disease, unspecified, with heart failure and with chronic kidney disease stage I through stage IV, or unspecified	Unspecified
404.93	Hypertensive heart and chronic kidney disease, unspecified, with heart failure and chronic kidney disease stage V or end stage renal disease	Unspecified
428	Heart failure	Unspecified
428.0	Congestive heart failure, unspecified	Unspecified
428.1	Left heart failure	Unspecified
428.2	Systolic heart failure	HFrEF
428.20	Systolic heart failure, unspecified	HFrEF
428.21	Acute systolic heart failure	HFrEF
428.22	Chronic systolic heart failure	HFrEF
428.23	Acute on chronic systolic heart failure	HFrEF
428.3	Diastolic heart failure	HFpEF
428.30	Diastolic heart failure, unspecified	HFpEF
428.31	Acute diastolic heart failure	HFpEF
428.32	Chronic diastolic heart failure	HFpEF
428.33	Acute on chronic diastolic heart failure	HFpEF
428.4	Combined systolic and diastolic heart failure	HFrEF
428.40	Combined systolic and diastolic heart failure, unspecified	HFrEF
428.41	Acute combined systolic and diastolic heart failure	HFrEF
428.42	Chronic combined systolic and diastolic heart failure	HFrEF
428.43	Acute on chronic combined systolic and diastolic heart failure	HFrEF
428.9	Heart failure, unspecified	Unspecified

© 2024 Vohra AS et al. JAMA Network Open.

eTable 2. Diagnosis Codes of Comorbid Conditions

Comorbid Condition	Methodology	Notes
Atrial fibrillation	CCW Chronic Conditions Algorithms	
Dementia	CCW Chronic Conditions Algorithms	1-year look-back
Metastatic cancer	Elixhauser Comorbidity Index	
Chronic kidney disease	CCW Chronic Conditions Algorithms	1-year look-back
COPD and Bronchitis	CCW Chronic Conditions Algorithms	
Depression	CCW Chronic Conditions Algorithms	
Diabetes	CCW Chronic Conditions Algorithms	1-year look-back
Ischemic Heart Disease	CCW Chronic Conditions Algorithms	
Hypertension	CCW Chronic Conditions Algorithms	
Peripheral vascular disease	Elixhauser Comorbidity Index	
Liver disease	Elixhauser Comorbidity Index	

© 2024 Vohra AS et al. JAMA Network Open.

eTable 3: Differences in Age and Sex Between Medicare Fee-For-Service and Medicare Advantage Populations in the Medicare 5% Sample

	Medicare Fee-For-Service						Medicare Advantage					
				Age Categories						Age Categories		
	N, % of bene	Men	Mean Age	65-74	75-84	85+	N, % of bene	Men	Mean Age	65-74	75-84	85+
2008	1682487 (75.33%)	39.5%	76.06	48.9%	33.7%	17.3%	551131 (24.67%)	37.9%	75.78	48.7%	36.5%	14.7%
2009	1673601 (73.86%)	39.9%	76.04	49.3%	33.1%	17.6%	592225 (26.14%)	38.5%	75.67	49.6%	35.8%	14.7%
2010	1685104 (73.26%)	40.3%	76.03	49.7%	32.6%	17.7%	614957 (26.74%)	38.9%	75.65	49.9%	35.4%	14.7%
2011	1711934 (72.65%)	40.8%	75.87	50.7%	31.7%	17.6%	644329 (27.35%)	39.2%	75.58	50.6%	34.7%	14.7%
2012	1731464 (71.24%)	41.4%	75.66	52.0%	30.6%	17.3%	698861 (28.76%)	39.7%	75.43	51.8%	33.6%	14.5%
2013	1744126 (69.74%)	41.9%	75.51	53.1%	29.8%	17.0%	756675 (30.26%)	39.9%	75.32	52.8%	32.8%	14.4%
2014	1745176 (67.86%)	42.4%	75.33	54.5%	28.9%	16.6%	826545 (32.14%)	40.0%	75.30	53.4%	32.2%	14.4%
2015	1769355 (66.98%)	42.7%	75.15	55.6%	28.3%	16.1%	872069 (33.02%)	40.3%	75.26	53.9%	31.8%	14.3%
2016	1805417 (66.46%)	43.0%	75.01	56.5%	27.9%	15.6%	911074 (33.54%)	40.5%	75.23	54.3%	31.6%	14.2%

© 2024 Vohra AS et al. JAMA Network Open.

**2017**	1819769 (64.98%)	43.4%	74.88	57.2%	27.8%	15.0%	980913 (35.02%)	40.7%	75.23	54.0%	31.8%	14.2%
**2018**	1834776 (63.41%)	43.7%	74.74	57.9%	27.7%	14.4%	1058754 (36.59%)	40.9%	75.21	54.0%	32.0%	14.0%
**2019**	1842494 (61.92%)	44.0%	74.68	58.4%	27.6%	14.0%	1133095 (38.08%)	41.1%	75.15	54.5%	31.9%	13.7%

© 2024 Vohra AS et al. JAMA Network Open.

eTable 4. Annual Numbers of Incident HF Patients

**Year**	**2008**	**2009**	**2010**	**2011**	**2012**	**2013**	**2014**	**2015**	**2016**	**2017**	**2018**	**2019**
Total	58982	58080	55656	53283	50061	50818	51907	51557	48520	52905	52242	50209
HFrEF	17712 (0.300)	20554 (0.354)	21408 (0.385)	21653 (0.406)	20772 (0.415)	22065 (0.434)	23510 (0.453)	24454 (0.474)	23555 (0.485)	24834 (0.469)	23960 (0.459)	22585 (0.450)
HFpEF	14367 (0.244)	17320 (0.298)	18958 (0.341)	19918 (0.374)	20318 (0.406)	21442 (0.422)	22579 (0.435)	23473 (0.455)	24304 (0.501)	26638 (0.504)	26853 (0.514)	26445 (0.527)
Un-specified	26903 (0.456)	20206 (0.348)	15290 (0.275)	11712 (0.220)	8971 (0.179)	7311 (0.144)	5818 (0.112)	3630 (0.0704)	661 (0.0136)	1433 (0.0271)	1429 (0.0274)	1179 (0.0235)

© 2024 Vohra AS et al. JAMA Network Open.

eTable 5: Risk-Adjusted Mortality Ratios, Relative to 2008, for Patients Hospitalized With Incident Heart Failure (2008-2018)

Time Increments	Acute (In-Hospital)	Post-Acute (0-30 Days)	Short-Term (31 Days- 1 Year)	Intermediate - Term (1-2 Years)	Long-Term (2- 3 Years)
2008	1.00 (0.99, 1.03)	1.00 (0.99, 1.01)	1.00 (0.99, 1.01)	1.00 (0.99, 1.01)	1.00 (0.99, 1.01)
2009	1.00 (0.97, 1.03)	0.99 (0.97, 1.01)	1.01 (1.00, 1.02)	1.02 (1.01, 1.04)	1.01 (1.00, 1.023)
2010	0.96 (0.93, 0.99)	0.99 (0.97, 1.01)	1.03 (1.02, 1.04)	1.02 (1.01, 1.03)	1.02 (1.01, 1.04)
2011	0.96 (0.93, 0.99)	1.01 (0.99, 1.03)	1.03 (1.02, 1.03)	1.05 (1.04, 1.07)	1.01 (1.00, 1.02)
2012	0.95 (0.92, 0.98)	1.01 (0.99, 1.03)	1.05 (1.04, 1.06)	1.02 (1.00, 1.03)	1.04 (1.02, 1.06)
2013	0.93 (0.90, 0.96)	1.05 (1.03, 1.07)	1.04 (1.03, 1.05)	1.06 (1.05, 1.07)	1.02 (1.01, 1.04)
2014	0.88 (0.85, 0.90)	1.00 (0.98, 1.03)	1.05 (1.04, 1.05)	1.03 (1.01, 1.04)	1.00 (0.99, 1.02)
2015	0.85 (0.83, 0.88)	1.00 (0.98, 1.02)	1.02 (1.01, 1.03)	1.01 (1.00, 1.03)	1.00 (0.99, 1.02)
2016	0.78 (0.75, 0.81)	0.96 (0.94, 0.98)	1.00 (0.99, 1.01)	0.98 (0.97, 0.99)	0.95 (0.93, 0.96)
2017	0.77 (0.74, 0.80)	0.93 (0.91, 0.95)	0.97 (0.96, 0.98)	0.94 (0.92, 0.95)	
2018	0.74 (0.71, 0.76)	0.88 (0.86, 0.90)	0.94 (0.94, 0.95)		

© 2024 Vohra AS et al. JAMA Network Open.

eTable 6. Risk Adjusted Mortality Ratios for In-hospital, and Post-Discharge Time Increments Relative to 2008 For HFrEF Patients

Time Increments	In Hospital	0-30 Days	31 Days- 1 Year	1-2 Year	2- 3 Year
Mortality Ratios					
2008	1.002 (0.942, 1.062)	0.999 (0.957, 1.041)	0.999 (0.982, 1.016)	1.000 (0.975, 1.024)	1.000 (0.973, 1.027)
2009	0.983 (0.928, 1.037)	1.088 (1.048, 1.128)	1.007 (0.992, 1.022)	1.020 (0.999, 1.041)	1.009 (0.983, 1.035)
2010	1.018 (0.965, 1.071)	1.103 (1.064, 1.142)	1.038 (1.022, 1.054)	1.019 (0.997, 1.040)	1.023 (0.996, 1.049)
2011	1.060 (1.006, 1.113)	1.152 (1.113, 1.190)	1.032 (1.017, 1.047)	1.051 (1.029, 1.072)	1.023 (0.999, 1.048)
2012	1.094 (1.037, 1.150)	1.143 (1.104, 1.181)	1.052 (1.037, 1.067)	1.021 (0.999, 1.042)	1.049 (1.023, 1.075)
2013	1.073 (1.018, 1.127)	1.189 (1.151, 1.227)	1.058 (1.044, 1.073)	1.069 (1.047, 1.091)	1.025 (1.000, 1.050)
2014	1.041 (0.991, 1.091)	1.135 (1.098, 1.173)	1.064 (1.050, 1.077)	1.024 (1.003, 1.045)	0.991 (0.967, 1.016)
2015	1.058 (1.008, 1.108)	1.143 (1.107, 1.179)	1.036 (1.022, 1.050)	0.994 (0.974, 1.014)	0.982 (0.960, 1.005)
2016	1.001 (0.953, 1.049)	1.127 (1.091, 1.164)	1.010 (0.997, 1.024)	0.982 (0.963, 1.001)	0.938 (0.915, 0.960)
2017	1.005 (0.959, 1.051)	1.106 (1.072, 1.140)	0.981 (0.968, 0.995)	0.927 (0.908, 0.946)	
2018	0.918 (0.873, 0.962)	1.044 (1.010, 1.078)	0.968 (0.956, 0.980)		

© 2024 Vohra AS et al. JAMA Network Open.

eTable 7. Risk Adjusted Mortality Ratios for In-hospital, and Post-Discharge Time Increments Relative to 2008 For HFpEF Patients

Time Increments	In Hospital	0-30 Days	31 Days- 1 Year	1-2 Year	2- 3 Year
Mortality Ratios					
2008	1.002(0.931, 1.072)	0.996(0.950, 1.043)	0.999(0.980, 1.018)	0.999(0.974, 1.024)	1.000(0.971, 1.029)
2009	1.129(1.060, 1.199)	1.000(0.959, 1.040)	1.037(1.019, 1.054)	1.064(1.041, 1.087)	0.984(0.958, 1.010)
2010	1.063(0.999, 1.126)	1.037(0.997, 1.077)	1.069(1.053, 1.085)	1.031(1.008, 1.053)	1.011(0.987, 1.036)
2011	1.101(1.043, 1.160)	1.052(1.013, 1.090)	1.058(1.041, 1.075)	1.092(1.069, 1.114)	0.980(0.956, 1.003)
2012	1.085(1.025, 1.144)	1.074(1.036, 1.112)	1.093(1.078, 1.109)	1.050(1.028, 1.072)	1.030(1.007, 1.053)
2013	1.046(0.986, 1.105)	1.132(1.094, 1.170)	1.074(1.059, 1.089)	1.097(1.076, 1.118)	1.009(0.986, 1.032)
2014	1.003(0.948, 1.057)	1.093(1.057, 1.129)	1.083(1.068, 1.097)	1.069(1.048, 1.089)	1.011(0.989, 1.034)
2015	0.979(0.926, 1.032)	1.094(1.058, 1.129)	1.068(1.055, 1.082)	1.069(1.049, 1.090)	1.011(0.988, 1.033)
2016	0.915(0.865, 0.964)	1.040(1.006, 1.073)	1.053(1.040, 1.066)	1.021(1.002, 1.039)	0.951(0.930, 0.972)
2017	0.848(0.804, 0.892)	0.996(0.966, 1.027)	1.022(1.009, 1.035)	0.986(0.969, 1.003)	
2018	0.861(0.816, 0.905)	0.941(0.912, 0.971)	0.983(0.971, 0.995)		

© 2024 Vohra AS et al. JAMA Network Open.

eFigure 1: Cumulative Risk-Adjusted Mortality Ratios for Full HF Cohort

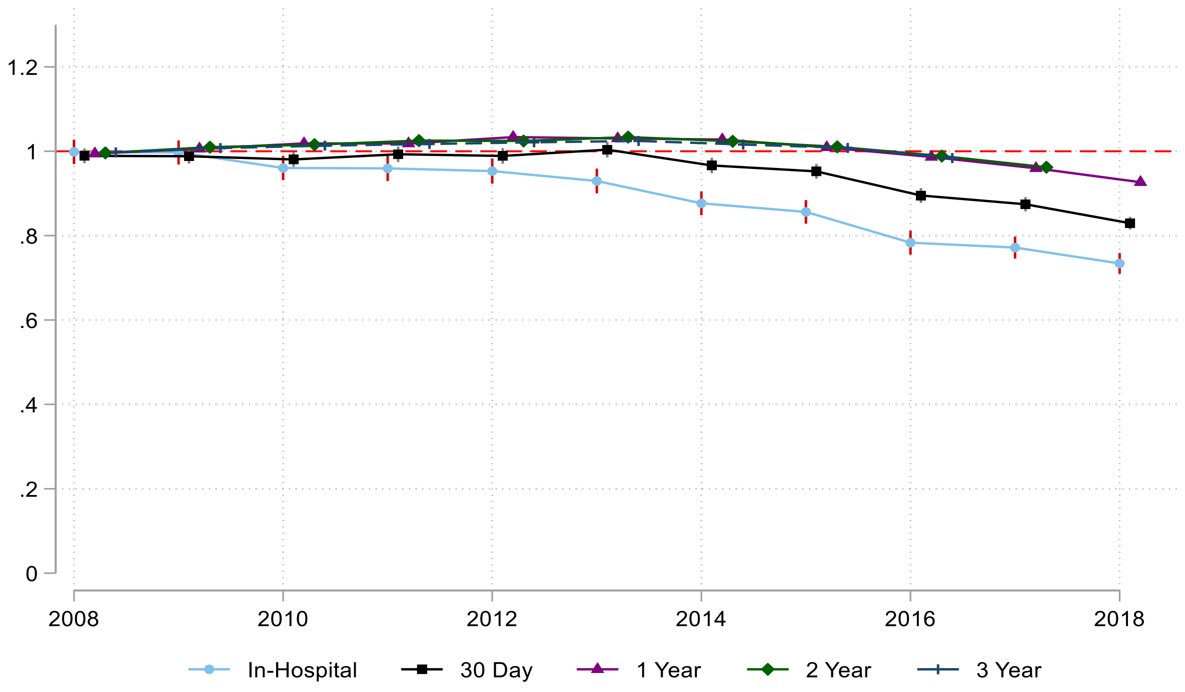

© 2024 Vohra AS et al. JAMA Network Open.

eFigure 2: Annual Proportion of Heart Failure Patients Classified as Reduced or Preserved Ejection Fraction

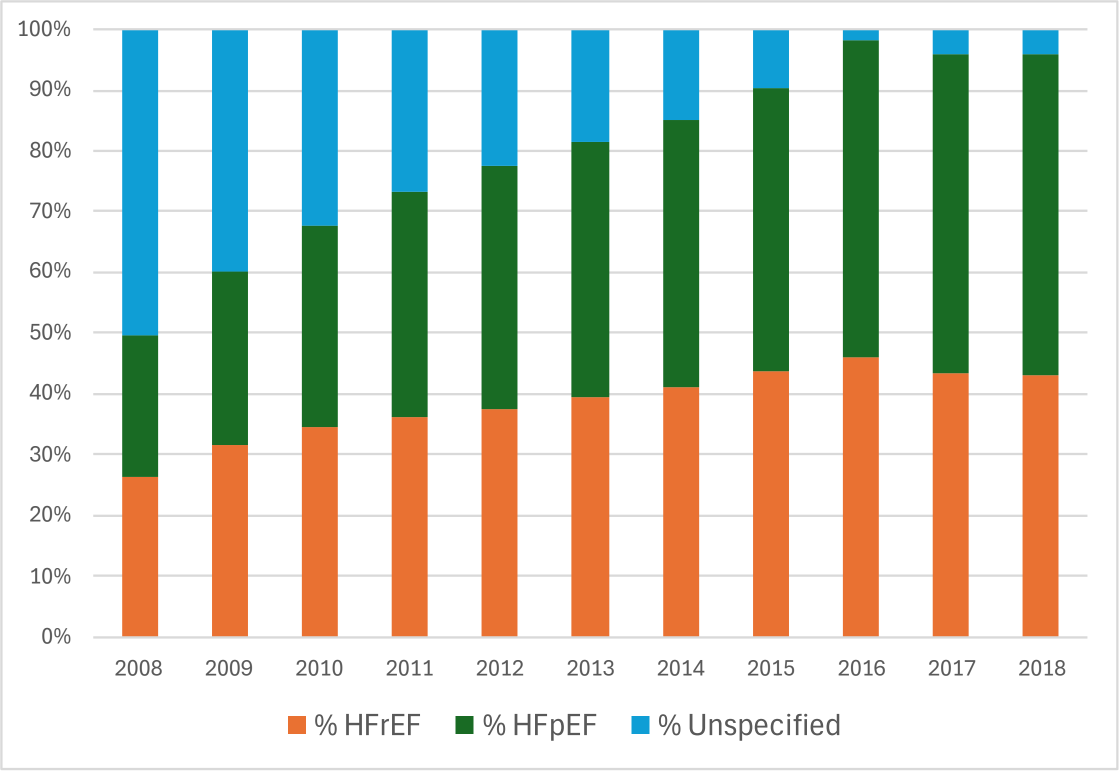

© 2024 Vohra AS et al. JAMA Network Open.

eFigure 3: Risk-Adjusted Mortality Ratios, Excluding Patients with Prior Outpatient HF Diagnosis (sensitivity analysis 1)

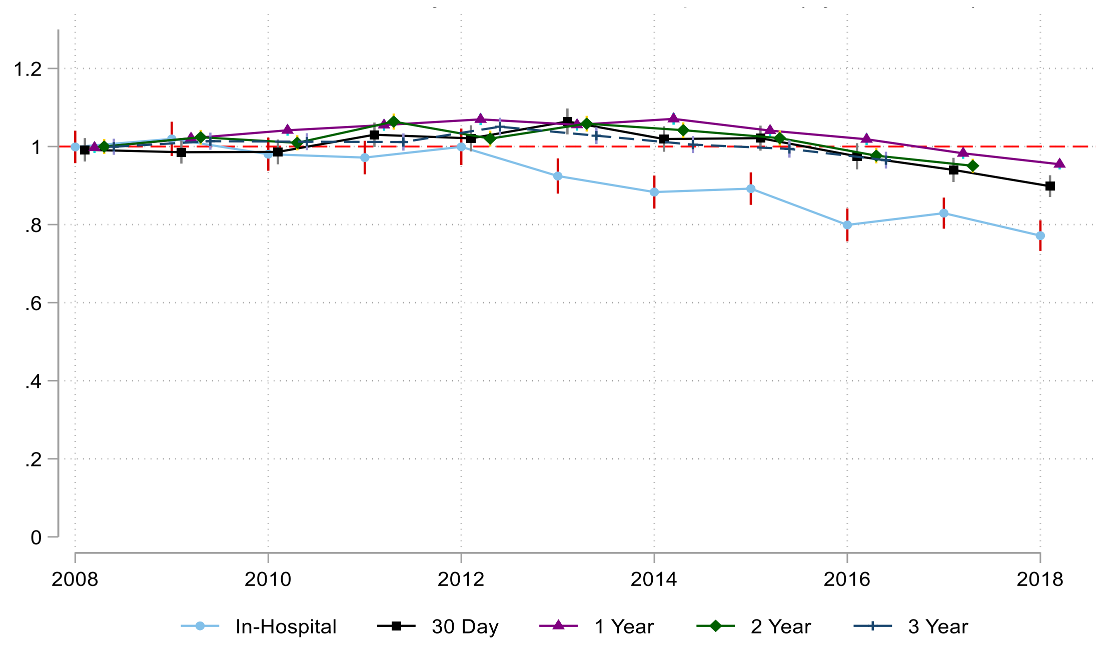

© 2024 Vohra AS et al. JAMA Network Open.

eFigure 4: **Risk-Adjusted Mortality Ratios, Including Length of Stay as Covariate (sensitivity analysis 2)**

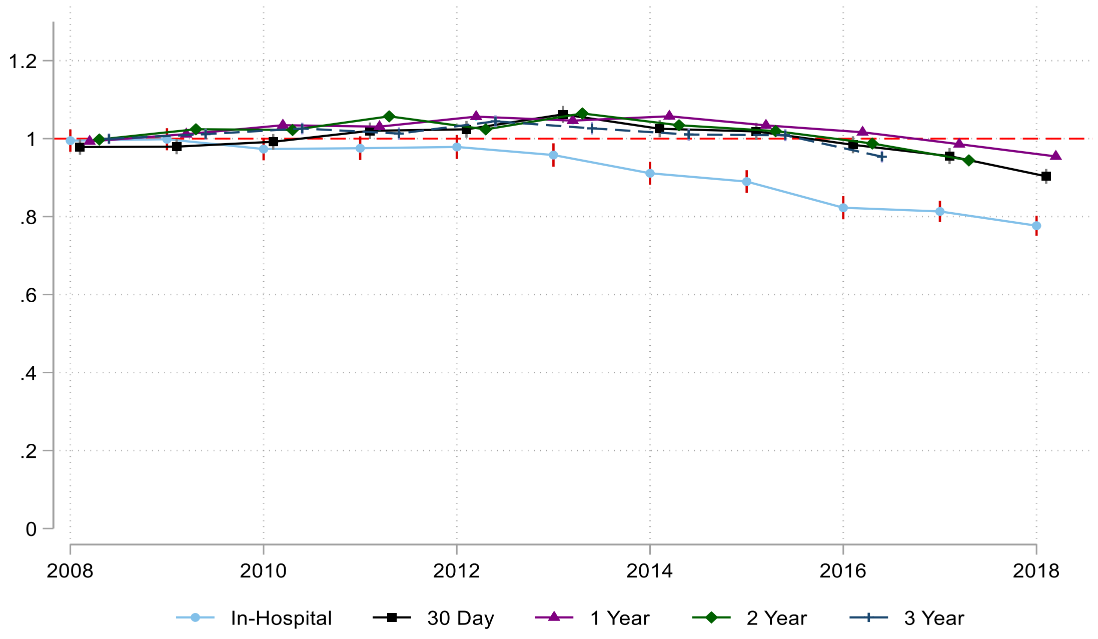

© 2024 Vohra AS et al. JAMA Network Open.

eFigure 5. Risk Adjusted Mortality Ratios for the Alternative Adjustment Methodology (sensitivity analysis 3)

Panel A: All HF

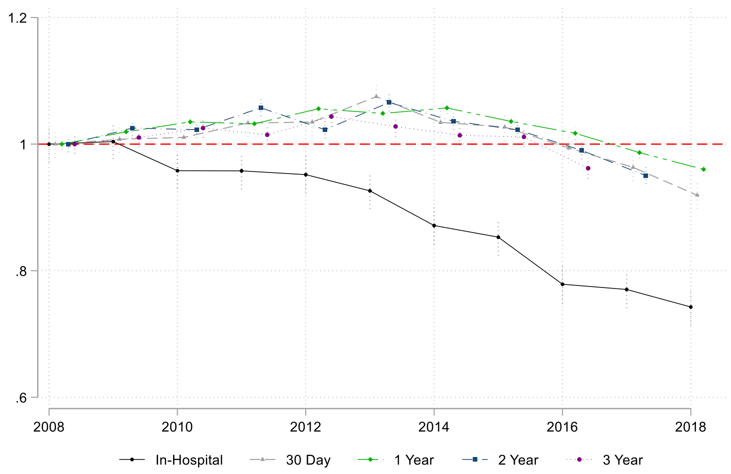

Panel B: HFrEF

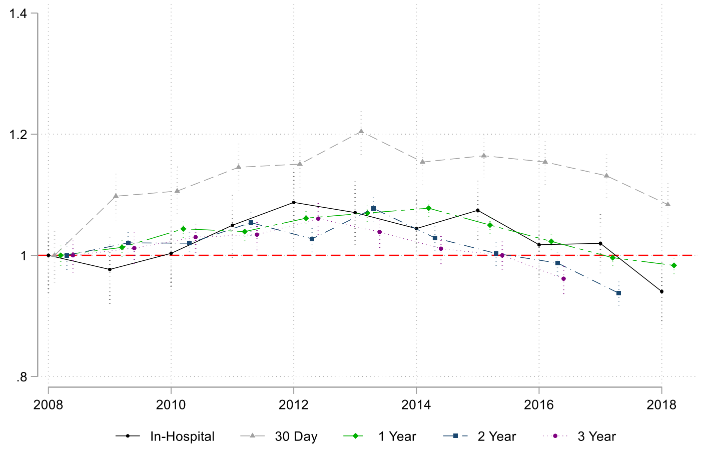

Panel C: HFpEF

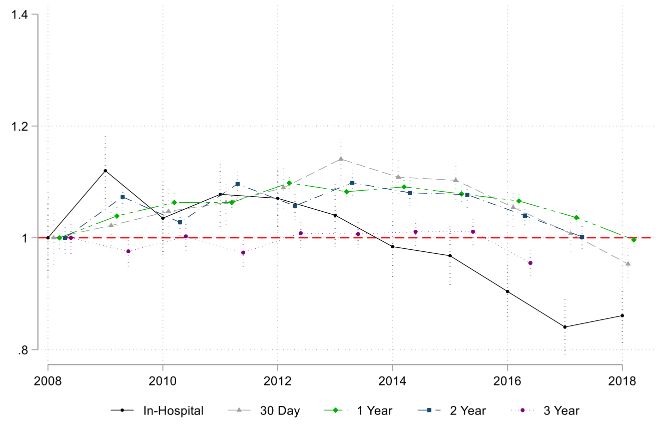

© 2024 Vohra AS et al. JAMA Network Open.
