# Supplementary material for: Trends in Mortality After Incident Hospitalization for Heart Failure Among Medicare Beneficiaries
Source: JAMA Netw Open. 2024 Aug 19;7(8):e2428964. doi: 10.1001/jamanetworkopen.2024.28964 (PMC11333983; doi:10.1001/jamanetworkopen.2024.28964)
Supplement: Supplement 2. — Data Sharing Statement [file jamanetwopen-e2428964-s002.pdf]

Data Sharing Statement

Vohra. Trends in Mortality After Incident Hospitalization for Heart Failure Among Medicare Beneficiaries. *JAMA Netw Open*. Published August 19, 2024.
doi:10.1001/jamanetworkopen.2024.28964

Data
Data available: No
